# Increased Phospholipid Transfer Protein Activity Is Associated With Markers of Enhanced Lipopolysaccharide Clearance in Human During Cardiopulmonary Bypass

**DOI:** 10.3389/fcvm.2021.756269

**Published:** 2021-10-12

**Authors:** Maxime Nguyen, Thomas Gautier, Guillaume Reocreux, Gaëtan Pallot, Guillaume Maquart, Pierre-Alain Bahr, Annabelle Tavernier, Jacques Grober, David Masson, Belaid Bouhemad, Pierre-Grégoire Guinot

**Affiliations:** ^1^Department of Anesthesiology and Intensive Care, Dijon University Hospital, Dijon, France; ^2^University of Burgundy and Franche-Comté, LNC UMR1231, Dijon, France; ^3^INSERM, LNC UMR1231, Dijon, France; ^4^FCS Bourgogne-Franche Comté, LipSTIC LabEx, Dijon, France; ^5^AgroSup, LNC UMR1231, Dijon, France

**Keywords:** endotoxemia, inflammation, lipopolysaccharide, lipoprotein, phospholipid transfer protein (PLTP), cardiopulmonary bypass

## Abstract

**Introduction:** Lipopolysaccharide (LPS) is a component of gram-negative bacteria, known for its ability to trigger inflammation. The main pathway of LPS clearance is the reverse lipopolysaccharide transport (RLT), with phospholipid transfer protein (PLTP) and lipoproteins playing central roles in this process in experimental animal models. To date, the relevance of this pathway has never been studied in humans. Cardiac surgery with cardiopulmonary bypass is known to favor LPS digestive translocation. Our objective was to determine whether pre-operative PLTP activity and triglyceride or cholesterol-rich lipoprotein concentrations were associated to LPS concentrations in patients undergoing cardiac surgery with cardiopulmonary bypass.

**Methods:** A *post-hoc* analysis was conducted on plasma samples obtained from patients recruited in a randomized controlled trial.Total cholesterol, high density lipoprotein cholesterol (HDLc), low density lipoprotein cholesterol (LDLc), triglyceride and PLTP activity were measured before surgery. LPS concentration was measured by mass spectrometry before surgery, at the end of cardiopulmonary bypass and 24 h after admission to the intensive care unit.

**Results:** High PLTP activity was associated with lower LPS concentration but not with inflammation nor post-operative complications. HDLc, LDLc and total cholesterol were not associated with LPS concentration but were lower in patients developing post-operative adverse events. HDLc was negatively associated with inflammation biomarkers (CRP, PCT). Triglyceride concentrations were positively correlated with LPS concentration, PCT and were higher in patients with post-operative complications.

**Conclusion:** Our study supports the role of PLTP in LPS elimination and the relevance of RLT in human. PLTP activity, and not cholesterol rich lipoproteins pool size seemed to be the limiting factor for RLT. PLTP activity was not directly related to post-operative inflammation and adverse events, suggesting that LPS clearance is not the main driver of inflammation in our patients. However, HDLc was associated with lower inflammation and was associated with favorable outcomes, suggesting that HDL beneficial anti-inflammatory effects could be, at least in part independent of LPS clearance.

## Introduction

Lipopolysaccharides (LPS; endotoxins) are one of the most studied pathogen-associated molecular patterns (PAMPs). They are components of the outer membrane of gram-negative bacteria, and they produce a harmful effect through the uncontrolled triggering of inflammation (1). During endotoxemia, LPS that are present in the blood can be recognized by the innate immune cells, mostly via the toll-like receptor-4 (TLR-4) (2) which initiates the inflammatory cascade. In patients with bacterial infection, LPS binding to TLR-4 triggers the immune response that is essential to fight against the pathogen. However, uncontrolled TLR-4 activation is deleterious and leads to sepsis and septic shock. Cardiac surgery with cardiopulmonary bypass triggers systemic inflammation, without pathogen involved. This sterile inflammation might also induce organ injury (3). Because endotoxemia is one of the mechanisms that triggers inflammation in cardiac surgery, reducing the endotoxin burdens might improve outcomes. On the opposite, and counteracting endotoxemia, the lipopolysaccharide reverse transport has been described by analogy with the reverse cholesterol transport (4). This pathway involves circulating lipoproteins which can bind and carry LPS to the liver where it is detoxified and eliminated through biliary secretion (4). One key player in this process is the plasma phospholipid transfer protein (PLTP) which promotes the binding LPS to lipoproteins thereby reducing their capacity to activate inflammation (5). This pathway thus modulates inflammation by preventing TLR4 activation.

PLTP is a member of the lipid transfer / LPS binding protein (LT/LBP) family. PLTP is an ubiquitous molecule. Active PLTP is mostly found associated with HDL in plasma (6). PLTP was first described for its role in phospholipid transfer between lipoproteins and its impact on HDL remodeling and metabolic fate. However, it has been demonstrated that PLTP can also bind LPS and transfer these molecules to lipoproteins (7), and recent reports show that PLTP can modulate the noxious effect of LPS and enhance LPS clearance in animal models (8, 9). Interestingly, high plasma PLTP activity was reported in septic patients as well as in experimental endotoxemia in humans (10), and increased PLTP levels could also be observed in a mouse model of infection (11). However, the relationship between PLTP levels and LPS concentration or clearance in humans is still unknown.

Sepsis occurs as the result of various pathological states, and patients with sepsis are admitted to hospital at different degrees of severity of the disease. Consequently, endotoxemia is inconstant in septic patients, and LPS plasma concentrations are highly variable depending on the cohort studied and the sampling pattern implemented (12). Overall, the exploration of pathological pathway such as reverse lipopolysaccharide transport is difficult in patients with sepsis. Cardiopulmonary bypass (CPB) is another condition that induce gut barrier dysfunction and LPS translocation (13). During cardiopulmonary bypass, endotoxemia has been reported as one of the factors that triggers systemic inflammation (14). Indeed, endotoxemia has been detected in the blood of patients undergoing cardiac surgery with CPB during the perioperative period and is associated with adverse outcomes (15, 16). On the contrary to naturally occurring sepsis, cardiopulmonary bypass (CPB) is a standardized procedure that provides a more reproducible model of acute inflammation. Furthermore, because surgery is planned, the time of injury is known. Baseline status can easily be determined, and the time to follow-up can be standardized.

We therefore set up a clinical study in patients with CPB to explore the role of lipoproteins and PLTP in LPS clearance by enhancing the reverse lipopolysaccharide transport pathway in humans. Our primary objective was to determine whether pre-operative PLTP activity and plasma lipid concentrations could be related to LPS clearance in patients undergoing cardiac surgery with CPB. Our secondary objectives were to determine whether PLTP activity and cholesterol concentrations were associated with inflammation and adverse outcomes.

## Materials and Methods

### Patients

This study is a *post-hoc* analysis of a prospective database. This database was created for a randomized control trial aiming to compare the effects of per and post-operative sedation either by sevoflurane or propofol on cardio protection (17). This database was approved by the institutional review board (CPP Est I, Dijon, France; ref. 2015-000476-99), and written, informed consent was obtained from all patients prior to surgery. Patients were prospectively included between October 2015 and August 2016. Inclusion criteria were: age ≥ 18 years, planned surgery with the use of CPB for coronary artery bypass grafting or the surgical correction of aortic stenosis. Exclusion criteria were: myocardial infarction <90 days prior to surgery, chronic renal failure with dialysis, pregnancy, occurrence of a serious adverse event, and withdrawal of consent. The present report was written in agreement with the STROBE statement (18).

### Protocol

Anesthesia, cardiopulmonary bypass and post-operative management were standardized for all patients as previously reported (17, 19). A plasma sample was taken at several time points: induction of anesthesia, end of CPB, and 24 h after admission to the intensive care unit (ICU). PLTP activity, total cholesterol concentration, and HDL and LDL cholesterol concentrations were retrospectively assayed at the pre-operative time point. LPS plasma concentrations, glucagon-like peptide-1 (GLP-1) and intestinal fatty acid binding protein (I-FABP) were retrospectively assayed at the three time points (16).

### Plasma Assays

LPS was quantifieded by liquid chromatography coupled with mass spectrometry. This method relies on the quantification of 3-hydroxymyristate (3HM, a fatty acid specific for the lipid A moiety of LPS). This method allows the complete quantification of LPS concentrations in plasma, as compared with an activity assay, which has the potential to omit a part of plasmatic LPS (i.e., inactive/neutralized LPS). The coefficient of variation of the method is 14% (20). All samples were processed from the same batch. In a cohort of 49 control subjects, the reported median 3HM concentration was 96 [77;116] pmol/ml (12).

Phospholipid transfer protein (PLTP) activity was measured using a commercially available fluorescence activity assay (Roar Biomedical Inc., New York) according to the manufacturer's instructions. Fluorescence measurements were performed over time on a Vicor^2^ multilabel counter (Perkin Elmer). Phospholipid transfer activity was calculated from the slope of fluorescence increase between 1 and 30 min.

Total cholesterol, LDL-cholesterol, HDL-cholesterol and triglyceride plasma concentrations were measured by commercially available enzymatic assays using the Thermo Fisher Scientific Indiko Clinical Chemistry Analyzer (Thermo Fisher).

### Endpoints

The primary endpoint was plasma LPS concentration. Perioperative plasma LPS concentrations refer to LPS plasma concentrations at all three time points. The secondary clinical endpoints were: a composite score of inflammation-related complications within 7 days (atrial fibrillation, acute kidney injury, stroke, and death), and ICU length of stay. Acute kidney injury was diagnosed according to the KDIGO definition (21). Secondary biological endpoints were: lactatemia after CPB, inflammation-related effects assessed by growth differentiation factor 15 (GDF-15) levels 24 h after admission, procalcitonin (PCT) and C-reactive protein (CRP) levels at admission, gastrointestinal disturbances assessed by: liver enzymes at admission, I-FABP and LPS after CPB, GLP-1 at 24 h after ICU admission, and myocardial damage assessed by troponin levels 6 h after ICU admission.

### Statistical Analysis

Normality was assessed with a Shapiro-Wilk test. Accordingly, quantitative data were presented as medians (interquartile range) or means (standard deviation). Groups were compared with a Student's *t*-test, Wilcoxon rank sum test, chi-squared or Fisher's exact test, as appropriate. Correlations were evaluated with the Spearman method. Longitudinal data were processed with mixed linear modeling. The association between PLTP, LDL cholesterol, HDL cholesterol, total cholesterol pre-operative concentrations and perioperative LPS concentrations were analyzed using multivariate mixed linear modeling. We first conducted an univariate analysis to assess the association between peri-operative LPS and the explanatory variable (including lipid parameters). Then we conducted a multivariate analysis. The lipid parameters were kept in the model (parameters of interests). Physiologically relevant variables with *p* < 0.15 by univariate analysis were considered as confounders for the multivariate analysis and included in the original model. Variable selection was automatized, backward, and *p*-value based, and the variables of interest were kept in the model. The critical *p*-value was set at 0.15. Multi-collinearity was addressed by coefficient correlations. The normality of the distribution of random effects and of the model residual were graphically checked. Because this analysis was exploratory, slight disparities between those distributions and normality were tolerated. Multivariate analysis inferences were checked on the log scale. There were three missing values for lipid parameters and PLTP activity, and five instances of missing data for LPS concentration, spread over the three sampling times. Missing data were considered at random and were omitted (in the bivariate analysis) or handled by the mixed model. The threshold for statistical significance was set at 0.05.

## Results

### Baseline Characteristics

Seventy-two patients were included in the present analysis. Median age was 70 [62;77] years and median BMI was 27.6 [25.3;30.1] kg/m^2^. A large majority of patients presented with hypertension (79.2%) and/or dyslipidemia (75.0%), and the median Euroscore 2 was 1.27 [0.83;2.09] (Table 1). 48.6% of patients were undergoing valve surgery, against 51.4% for coronary artery bypass graft surgery. Median duration of cardiopulmonary bypass was 89.5 [78.5;113] min.

**Table 1:** Baseline characteristics.

	**All *N* = 72**
Age (years)	70 [62;77]
BMI (kg/m^2^)	27.6 [25.3;30.1]
Women *n* (%)	24 (33.3%)
Euroscore II (%)	1.27 [0.83;2.09]
**Medical history**	
Hypertension *n* (%)	57 (79.2%)
Diabetes *n* (%)	26 (36.1%)
Dyslipidemia *n* (%)	54 (75.0%)
Active smoking *n* (%)	10 (13.9%)
Stroke *n* (%)	7 (9.72%)
Myocardial infarction *n* (%)	14 (19.4%)
LVEF (%)	61.1 (9.11)
Estimated glomerular filtration rate (ml/min/1.73 m^2^)	79.4 (17.6)
**Treatment** ***n*** **(%)**	
Beta blockers	43 (59.7%)
Statins	50 (69.4%)
Diuretics	25 (34.7%)
Oral antidiabetic agent	21 (29.2%)
Angiotensin converting enzyme inhibitor	52 (72.2%)
Calcium channel blocker	19 (26.4%)
**Surgery**	
Aortic valve surgery *n* (%)	35 (48.6%)
Coronary artery bypass graft surgery *n* (%)	37 (51.4%)
**Lipid parameters (plasma concentration)**	
PLTP activity (A.U.)	127.0 (±51.4)
Total cholesterol (g/L)	1.46 [1.09;1.76]
Total cholesterol (mmol/L)	3.78 [2.82;4.55]
HDLc (g/L)	0.37 [0.30;0.46]
HDLc (mmol/L)	0.96 [0.78;1.19]
LDLc (g/L)	0.61 [0.46;0.88]
LDLc (mmol/L)	1.58 [1.19;2.28]
Triglycerides (g/L)	1.14 [0.93;1.50]
Triglycerides (mmol/L)	1.29 [1.05; 1.69]

*Data are expressed as means ± standard deviation, medians (25^th^-75th percentiles), or numbers (%)*.

*BMI, body mass index; LVEF, Left ventricular ejection fraction*.

There were no differences of lipid parameters between obese and non-obese patients (all *p*-values > 0.05). As expected, diabetic patients had lower HDLc concentration (0.32 [0.27;0.40] vs. 0.39 [0.32:0.51] g/L; *p* < 0.01), lower LDLc concentration (0.50 [0.37;0.64] vs. 0.71 [0.52;0.95] g/L; *p* < 0.01) and higher triglyceride concentration (1.35 [1.04;1.69] vs. 1.12 [0.86;1.40] g/L; *p* = 0.02). PLTP activity did not significantly differ in diabetic patients. Baseline LPS concentration was higher in diabetic patients (132 [109;175] vs 110 [91;138] pmol/ml of 3HM; *p* = 0.02).

### Primary Outcome

**Figure 1:**
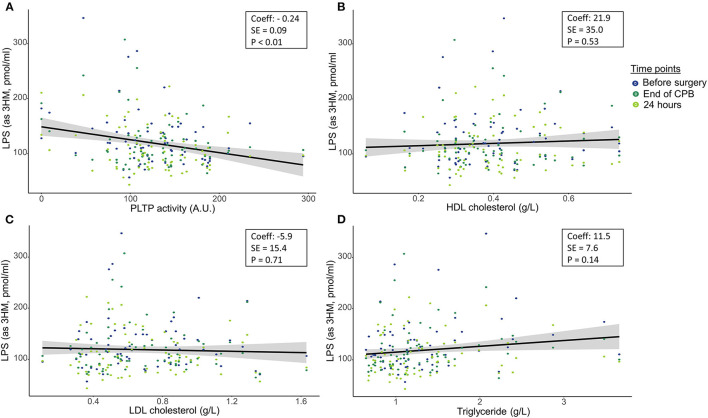
Association between lipopolysaccharide concentrations at the three perioperative time-points and pre-operative **(A)** phospholipid transfer protein, **(B)** HDL cholesterol, **(C)** LDL cholesterol and **(D)** Triglycerides. LPS, Lipopolysaccharide; 3HM, 3-hydroxymyristate; PLTP, phospholipid transfer protein; HDL, High density lipoprotein; LDL, low density lipoproteins. Plain lines are regression lines using the least squared methods. *P*-values were computed using mixed linear modeling to handle repeated measures. Coeff: Regression coefficient; SE: Standard error.

Median LPS concentration (expressed in pmol/ml of 3HM) was 118 [92;147] before surgery, 107 [92;131] at the end of CPB and 99 [79;133] 24 h after ICU admission. Mean pre-operative PLTP activity was 127.0 ± 51.4 A.U. Median HDL cholesterol (HDLc) was 0.37 [0.30;0.46] g/L, median LDL cholesterol (LDLc) was 0.61 [0.46;0.88] g/L and median triglyceride concentration was 1.14 g/L [0.93;1.50]. In univariate analyses, perioperative LPS concentrations were negatively associated with PLTP activity, but not with total cholesterol, HDLc or LDLc and triglyceride concentration (Figure 1). PLTP activity and triglyceride concentrations were associated with LPS concentrations in multivariate analysis (Table 2). The main identified confounders were stroke and pre-operative renal clearance.

**Table 2:** Variables associated with perioperative lipopolysaccharide concentrations in multivariate analysis.

	**Coeff (se)**	***P***
HDLc	40.0 (33.4)	0.24
LDLc	−15.9 (12.9)	0.22
Triglycerides	19.1 (6.5)	<0.01
PLTP activity	−0.17 (0.07)	0.02
History of stroke	74.0 (12.5)	<0.01
Preoperative GFR	−0.60 (0.21)	<0.01
**Time of sampling**		
End of CPB	−7.5 (5.0)	0.14
At 24 h	−16.5 (5.0)	<0.01

*Coeff, regression coefficient; SE, standard error; HDLc, HDL cholesterol; LDLc, LDL cholesterol; PLTP, phospholipid transfer protein; GFR, glomerular filtration rate; CPB, cardiopulmonary bypass*.

### Secondary Outcomes

The correlations between lipid parameters, general markers of surgical burden, of the biological markers of gastrointestinal dysfunction, and of inflammation are shown in Table 3. There was no association between lipid parameters and exposure to CPB, peak lactatemia or troponin (all *p*-values > 0.05). Regarding markers of gastrointestinal dysfunction, we found no association between PLTP activity, HDLc or total cholesterol with ALT, AST, I-FABP, or GLP-1 (all *p*-values > 0.05). LDLc was only associated with GLP-1 24 h after ICU admission.

**Table 3:** Association between lipid parameters and secondary outcomes.

	**PLTP activity**	**Total cholesterol**	**HDLc**	**LDLc**	**Triglycerides**
**Surgical burden**					
Lactates at the end of CPB	−0.12	0.02	−0.01	0.06	0.00
Duration of CPB	0.03	−0.16	−0.20	−0.18	0.14
Troponin at 6 h	−0.12	−0.07	−0.01	−0.05	−0.10
**Markers of GI dysfunction**					
ALT at ICU admission	0.06	−0.05	−0.21	−0.08	0.40^*^
AST at ICU admission	−0.09	−0.14	−0.14	−0.14	0.21
I-FABP after CPB	−0.11	0.03	−0.08	0.09	−0.09
LPS after CPB	−0.12	0.05	0.00	0.02	0.30^*^
GLP-1 at 24 h	−0.20	−0.22	−0.07	−0.24^*^	0.07
**Inflammation**					
CRP at ICU admission	0.17	−0.17	−0.46^*^	−0.18	0.15
PCT at ICU admission	0.09	−0.15	−0.26^*^	−0.20	0.28^*^
GDF-15 at 24 h	0.09	−0.02	0.04	−0.04	−0.01

*PLTP, phospholipid transfer protein; HDLc, HDL cholesterol; LDLc, LDL cholesterol; CPB, cardiopulmonary bypass; GI, gastrointestinal; ICU, intensive care unit; ALT, Alanin amino transferase; I-FABP, Intestinal fatty acid binding protein; GLP-1, glucagon-like binding protein*.

*Results are presented as spearman correlation coefficients*.

* *significant correlation (p < 0.05)*.

PLTP activity, total cholesterol and LDLc were not associated with any of the inflammatory biomarkers (see Table 3). High HDLc was correlated with lower post-operative CRP and PCT (r = −0.46 and −0.26, respectively, *p* < 0.05 in both cases).

**Figure 2:**
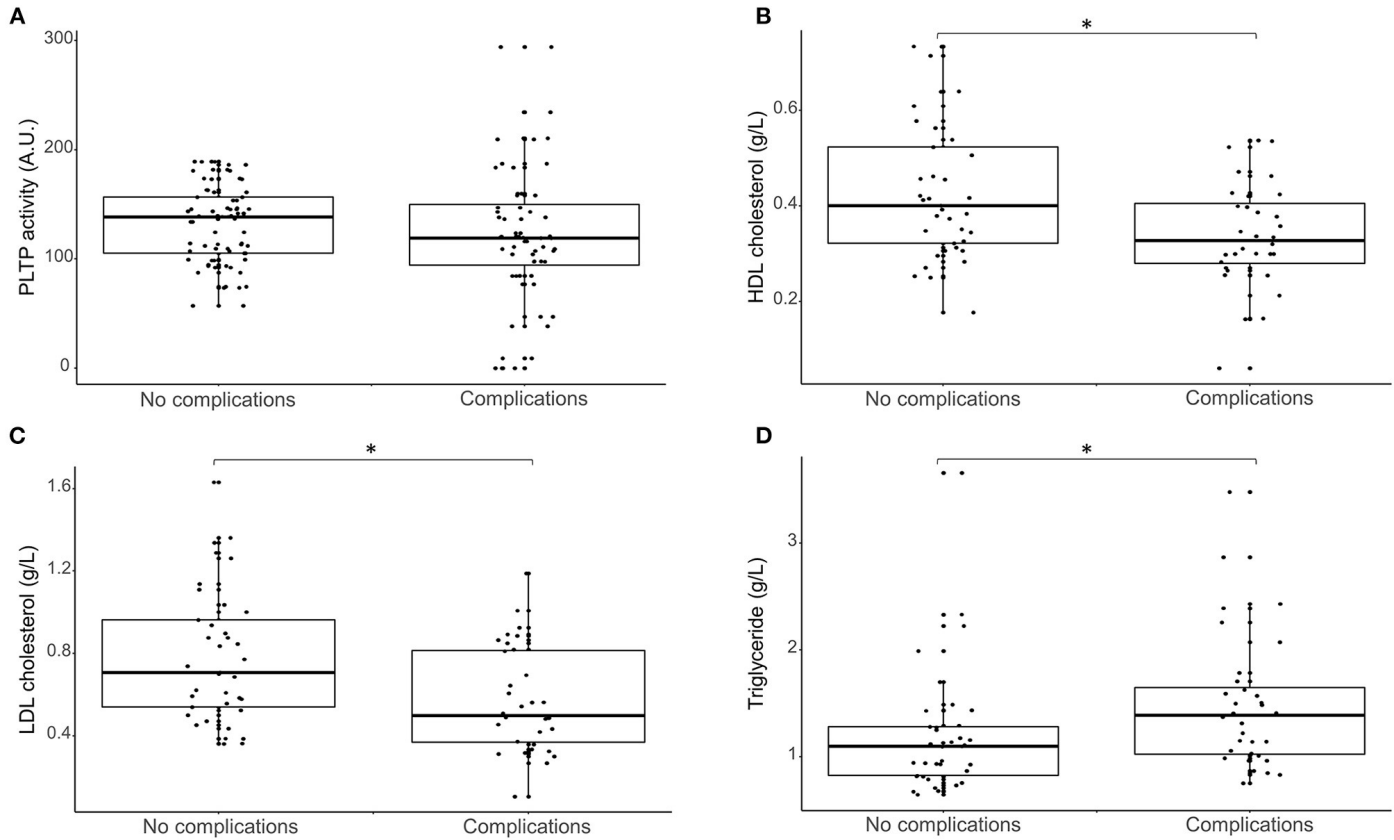
Box plot representing the association between lipid parameters (**A**: PLTP activity, **B**: HDL cholesterol concentration, **C**: LDL cholesterol concentration and **D**: Triglyceride concentration) at baseline and post-operative complications. PLTP, phospholipid transfer protein; HDL, High density lipoprotein; LDL, low density lipoproteins. *significant differences (*p*-value < 0.05).

Finally, patients were stratified according to composite clinical criteria and PLTP activity and lipid parameters were compared between the group with no complications and the group with complications (Figure 2). PLTP activity was not different for patients who developed the composite clinical criteria compared with the others (*p* = 0.43). Low HDLc (Figure 2B) and LDLc (Figure 2C) were associated with adverse outcomes (0.40 [0.32;0.52] vs. 0.33 [0.28;0.40] g/L *p* = 0.01 and 0.71 [0.54;0.96] vs. 0.50 [0.37;0.82] g/L; *p* = 0.01 respectively). On the opposite, triglycerides (Figure 2D) were higher in patients who experienced adverse clinical events (1.10 [0.82;1.28] vs. 1.39 [1.02;1.65] g/L; *p* < 0.01).

## Discussion

Our main finding is that high PLTP activity was associated with lower perioperative concentrations of LPS, but not with markers of gastrointestinal dysfunction, supporting that it has a role in LPS clearance in humans. However, PLTP was not associated with post-operative inflammation and clinical complications, suggesting that LPS pathway may not be the main driver for inflammation in this specific condition. Pre-operative HDLc was not associated with LPS concentration but was negatively associated with post-operative inflammatory markers (CRP, PCT) and clinical outcome, suggesting those beneficial anti-inflammatory effects to be independent of LPS clearance.

To the best of our knowledge, our study is the first to report an association between PLTP activity and LPS concentrations in humans. We also found that PLTP activity was not associated with any marker of gastrointestinal dysfunction, suggesting that the association between PLTP and LPS is not related to increased endotoxin translocation. Thus, in line with animal findings (8, 22), our results further confirm the implication of PLTP in LPS clearance. Because LPS is amphipathic, most LPS molecules are bound to lipoproteins, mostly HDL, in human blood (23–25). In our results, HDLc and LDLc were not associated with LPS concentration. Because in our population, only simple procedures were included (i.e., aortic valve surgery or coronary bypass), exposure to CPB and LPS burden were low (absence of post-operative increase in LPS concentration). In this setting, we hypothesized that the lipoprotein pool size was not a limiting factor for LPS clearance by the RLT pathway.

Despite its association with LPS concentrations, pre-operative PLTP activity was not associated with post-operative inflammation or clinical adverse outcomes. During CPB, many different factors trigger inflammation. Indeed, in addition to endotoxemia, activation of circulating blood elements interacting with artificial surfaces in the bypass circuit, shear forces induced by pump systems, hypothermia, ischemia reperfusion, surgical trauma (release of danger associated molecular patterns) have been reported to trigger inflammation (14, 26). In this context of low LPS burden, it is probable that LPS clearance is not the main protective mechanism against systemic inflammation. On the contrary, in Gram negative sepsis, LPS is one of the main driver for inflammation and the administration of key players of the RLT (i.e., HDL, PLTP) improved survival in animals models (9, 27). Altogether, even though the RLT did not have direct clinical implications in our population, this pathway might be a promising therapeutic target in other diseases influenced by the presence of LPS such as Gram-negative sepsis or atherosclerosis. Thus, demonstrating its relevance in human is a key step in this process.

Lipoproteins exert multiple effects against inflammation (28). Patients with sepsis have decreased concentrations of cholesterol rich lipoproteins and increased triglyceride concentrations (29) and lipid levels measured before the aggression have been related to adverse outcome. Indeed, in a recent study, high or low level of pre-sepsis LDL cholesterol and triglyceride concentrations were associated with increased mortality (30). In cardiac surgery, associations between cholesterol-rich lipoproteins, inflammation and adverse outcomes have previously been reported (31). Furthermore, pre-operative low cholesterol concentrations were associated with higher inflammation and clinical systemic inflammatory response syndrome (32). Furthermore, a randomized controlled trial, demonstrated that pre-operative administration of statin decrease LDL cholesterol and increase acute kidney injury (33). In our patients, HDLc was not associated with LPS concentration but was associated with lower inflammation and post-operative complications. Thus, it appeared that HDL exerted anti-inflammatory effects that were independent of LPS elimination. LDLc and triglyceride plasma concentration were also associated with post-operative complication.

Because patients are required to fast before undergoing cardiac surgery, triglyceride concentrations in our patients probably reflected the very low-density lipoprotein (VLDL) pool size. In our patients, high triglyceride concentrations were associated with LPS, PCT and adverse outcomes. Though triglycerides have been described as protective against inflammation and mortality in sepsis (30, 34), previous reports also suggest that baseline triglyceride concentrations are higher in patients who develop post-operative systemic inflammation (32). LPS has been described as increasing the synthesis and secretion of triglyceride-rich lipoproteins and reducing lipoprotein lipase activity (35). However, we performed triglyceride measurements prior to CPB. In this context, we hypothesized that hypertriglyceridemia might reflect a pre-operative condition that promotes gut barrier dysfunction and LPS translocation during CPB. In particular diabetes, i.e., a condition that is well-known for its association with low-grade inflammation and altered triglyceride metabolism (36), was associated with both hypertriglyceridemia and high baseline LPS concentration in our population.

There are some limitations that should be taken into consideration. The sample size (72 patients), limited the power of the analysis. Because this study was observational, associations can be made, but causality cannot be proven. The monocentric design and the selected population limit the external validity of our results. LPS activity could not be retrospectively assayed from frozen plasma, therefore the neutralization process could not be studied. Post-operative LPS concentrations did not increase, probably due to per-operative hemodilution and because albumin and protein concentration were not reported in the data base, we could not correct those concentrations. Because HDL size might vary (in particular might be modified by PLTP), HDLc might not precisely reflect HDL particle concentration (37). Finally, all patients received propofol, which contains a triglyceride emulsion, creating a potentially confounding condition. Subsequently, triglyceride baseline concentration are probably overestimated. However, the induction sequence wa protocolized and every patient received propofol, thus limiting its confounding effect in correlation and regression analysis.

In conclusion, our study supports the role of PLTP in LPS elimination and the relevance of RLT in humans. PLTP activity seemed to be the limiting factor for RLT rather than cholesterol-rich lipoprotein pool size. PLTP activity was not directly related to post-operative inflammation and adverse events, suggesting that LPS clearance is not the main driver of inflammation in cardiac surgery with cardiopulmonary bypass. On the contrary, HDLc was associated with lower inflammation and HDLc was associated with favorable outcomes, suggesting that the beneficial anti-inflammatory effects were, at least in part, independent of LPS clearance. PLTP and lipoprotein pool size might represent future therapeutic target against acute inflammation. However, their efficiency could depend on both the trigger and the intensity of the inflammatory process.

## Data Availability Statement

The raw data supporting the conclusions of this article will be made available by the authors, without undue reservation.

## Ethics Statement

This is an ancillary analysis of a randomized controlled trial. The studies involving human participants were reviewed and approved by CPP Est I, Dijon, France; ref. 2015-000476-99. The patients/participants provided their written informed consent to participate in this study.

## Author Contributions

MN, BB, TG, and P-GG analyzed the data and drafted the manuscript. GP, AT, JG, and GM performed the assay. DM, GR, P-AB, and GM reviewed and edited the manuscript. All the authors have read and approved the manuscript.

## Funding

This work was supported by a French Government grant managed by the French National Research Agency (Agence Nationale de la Recherche) as part of the Investissements d'Avenir program, reference ANR-11-LABX-0021-01- LipSTIC Labex. It is also part of two integrated projects funded by the European Union (FEDER) and the French government (Regional Council of Bourgogne-Franche-Comté).

## Conflict of Interest

The authors declare that the research was conducted in the absence of any commercial or financial relationships that could be construed as a potential conflict of interest.

## Publisher's Note

All claims expressed in this article are solely those of the authors and do not necessarily represent those of their affiliated organizations, or those of the publisher, the editors and the reviewers. Any product that may be evaluated in this article, or claim that may be made by its manufacturer, is not guaranteed or endorsed by the publisher.
